# Cardiac calcium score on 2D echo: correlations with cardiac and coronary calcium at multi-detector computed tomography

**DOI:** 10.1186/1476-7120-12-43

**Published:** 2014-10-28

**Authors:** Nicola Gaibazzi, Chiara Baldari, Pompilio Faggiano, Lisa Albertini, Giacomo Faden, Filippo Pigazzani, Cristina Rossi, Claudio Reverberi

**Affiliations:** Cardiology Department, Parma University Hospital, 43123 Parma, Italy; Radiology Department, Parma University Hospital, Parma, Italy; Cardiology Department, University of Brescia, Brescia, Italy

**Keywords:** Echocardiography, Valve, Coronary artery disease, Aortic valve, Mitral valve

## Abstract

**Background:**

To test the hypothesis that a semi-quantitative echocardiographic calcium score (eCS) significantly correlates with cardiac calcium measured by coronary computed tomography angiography (CCTA) and, secondarily, severe coronary artery calcifications and stenosis.

**Methods:**

This is a retrospective, observational study, conducted in a tertiary centre. eCS was compared with CCTA scores of non-coronary cardiac calcium (nCACS), coronary cardiac calcium (CACS) and number of diseased coronary vessels, in 141 subjects without known coronary artery disease (CAD), who underwent both echocardiography and CCTA for clinical reasons.

**Results:**

Age, prevalence of hypertension and all measures of calcium (eCS, nCACS and CACS) differed significantly between the no-CAD and CAD subgroups. eCS was positively correlated with nCACS (Spearman rho = 0.64, p < 0.0001), CACS (rho = 0.46, p < 0.01) and weakly with the number of diseased coronary vessels (rho = 0.28, p < 0.05). eCS and nCACS had similar area under the curve (AUC) for the prediction of severe CACS (≥400) (0.77, 95% CI 0.68-0.86 and 0.79, 95% CI 0.72-0.88) or obstructive CAD (0.63, 95% CI 0.54-0.72 and 0.63, 95% CI 0.55-0.73).

**Conclusions:**

eCS, a calcium score easily obtainable during standard echocardiography, is moderately to strongly correlated with nCACS by CCTA. The full eCS score correlates with nCACS better than its single components. It correlates with CACS and predicts severe coronary calcification (CACS > 400), a known predictor of cardiovascular morbidity and mortality. The eCS also predicts obstructive CAD, incrementally to age and clinical variables, although for this purpose CACS remains the most accurate score.

## Background

Coronary artery calcifications are strongly correlated with atherosclerosis and the coronary artery calcium score (CACS) by coronary computed tomography angiography (CCTA) can be used to make better prediction regarding the individual risk of future coronary events [1–4].

Using CCTA, the presence of aortic valve calcification (AVC) and mitral annulus calcification (MAC) has been associated with CACS [5, 6] presence, extent, and vulnerable characteristic of coronary plaque [7]. Similarly, AVC, MAC or other semi-quantitative scores of cardiac calcium using echocardiography have also been associated with coronary artery disease (CAD) and, more importantly, adverse cardiac prognosis [8–19].

Cardiac calcifications have been historically recognized since the early days of ultrasound imaging [20], but only recently the correlation between cardiac non-coronary calcium measured either by echocardiography or CCTA was investigated, in a small group of 41 subjects [21]. While CCTA remains the reference method to quantify coronary and non-coronary cardiac calcium, the use of echocardiography for this purpose, if validated against CCTA, would portend inherent advantages of low-cost, portability and radiation safety, becoming a potentially simple adjunct to clinical scores for individualized risk prediction.

To test the hypothesis that a semi-quantitative echocardiographic calcium score (eCS) significantly correlates with cardiac calcium measured by CCTA, we compared it with CCTA generated scores of non-coronary cardiac calcium (nCACS), and secondarily with CACS and number of diseased coronary vessels, in a wide cohort of subjects who underwent both echocardiography and CCTA for clinical reasons.

## Methods

### Study population

Patients who underwent a clinically indicated CCTA between January 2012 and September 2013 were identified. Patients with known CAD or previous coronary events or suspect of cardiac neoplastic mass as an indication were excluded. Patients with pacemaker leads, sternal wires or prosthetic valves were also excluded from the study because usually these are patients with a high prevalence of CAD or other heart disease.

Echocardiography database was then searched to identify those patients who also had an echocardiogram performed within 3 months of their CCTA scan. Both the echocardiograms and CCTA scans were read separately and independently by 2 different observers (LA, CB) who were unaware of the results of the other test.

Hypertension was defined as systolic blood pressure >140 mmHg and or diastolic pressure >90 mmHg or current use of antihypertensive medications. Diabetes mellitus was defined as a history of oral hypoglycaemic drugs or insulin use or fasting blood glucose levels ≥126 mg/dl. Tobacco use was defined as currently smoking cigarettes. Family history was defined as CAD in first degree relatives, in men <55 y/o and women <65 y/o. Hypercholesterolemia was defined as history of total cholesterol >200 mg/dl or use of cholesterol lowering drugs and obesity as a body mass index > 30.

### Computed tomography

The study was performed with a Definition Flash system (Siemens, Forchheim, Germany). This CT system has two X-ray tubes and two detector arrays rotating in the same plane with an angular offset of 95°. Gantry rotation time is 280 ms, which provides a temporal resolution of 75 ms using a heart rate independent single-segment reconstruction and high pitch up to 3.4. Detector collimation is 2 × 64 × 0.6 mm. A z-axis flying focal spot is applied which results in an acquisition of 2 × 128 slices per rotation.

Two scans were performed in all patients: one to visualise coronary artery calcium and one angiography scan.

**CACS:** First patients underwent nonenhanced prospective electrocardiography (ECG)-gated sequential scan to measure CACS. The corresponding images for calcium scoring were reconstructed with a slice width of 2.5–3 mm and slice interval of 1.25–1.5 mm and the tube voltage was 120 kVp. A region of interest was drawn over the areas of calcification and the Agatston score was automatically calculated by the software. A cutoff value above 130 Hounsfield units to define calcification and lesion area multiplied by a density factor derived from the maximal Hounsfield units [22]. Separate scores were calculated for the aortic root, aortic valve, mitral valve, papillary muscles, and for the coronary arteries. For the calculation of aortic root calcium, only calcifications extending up to 2 cm above the sinus of Valsalva were used, since this is the level most often imaged by transthoracic echocardiography. Any calcification, seen as a ring, or part of a ring at the aortic annulus was included in the aortic root and not as coronary or aortic valve calcium. Non-contrast scans used to assess CACS were compared with contrast-enhanced scans as well. Contrast-enhanced scans were reconstructed in multiple views.

**CTCA:** All scans started above the carina and extended to just below the diaphragm, including the entire coronary tree. Scans were done with breath held in inspiration.

A bolus of iodinated contrast material (Iomeron 400 Bracco Imaging SpA, Milan, Italy) was injected (flow rate, 6.0 mL/sec) followed by a saline chaser with the same flow rate. To synchronize acquisition of the coronary CTA dataset to arterial enhancement, a “test bolus” protocol was used. CTCA was performed with adaptive electrocardiographic (ECG) pulsing which preserves diagnostic image quality and performance. A prospective ECG-triggering high-pitch spiral mode or retrospective ECG gating spiral mode were used. All images were reviewed on a workstation (Leonardo Siemens) equipped with a dedicated software tool for calcium scoring (Calcium Scoring CT, Siemens), using multiple windows so that circumflex coronary artery calcification could be delineated from mitral annular calcification [21].

Effective dose was estimated based on the doe-length product, using a conversion factor of 0.014 for chest CT in adults. The estimated effective dose of prospectively ECG-triggered CTCA using this type of equipment has been evaluated in average approximately 1–2.5 mSv; if retrospective triggering is needed, dose is significantly higher, approximately 10–15 mSv [23].

### Echocardiography

All selected patients underwent a standard rest transthoracic echocardiography using Philips ie33 system equipped with S5 probe, within 3 months from CCTA. Criteria for judging AVC, MAC, Aortic root and papillary muscle calcium were similar to grading systems used in previous studies [9, 16] and are detailed in Table 1. AVC was defined as focal areas of increased echogenicity and thickening of the aortic valve leaflets in the absence of aortic stenosis (velocity across the valve <2.5 m/sec). Each aortic valve leaflet was graded on a scale of 0 (normal) to 3 (severe) according to leaflet thickening and calcific deposits; the highest score for a given cusp was assigned as the overall degree of aortic valve sclerosis. MAC was defined as an intense and bright echo-producing structure located at the junction of the atrioventricular groove and posterior mitral valve leaflet and was measured from the leading anterior to the trailing posterior edge and judged on a scale of 0 (normal) to 3 (severe). Papillary muscle calcium was defined as a bright echo involving the head of 1 or both papillary muscles. Aortic root calcium was defined as a focal or diffuse area of increased echoreflectance and thickening in the aortic root on the parasternal long-axis view. Accordingly, a final score was derived as the sum of all identified cardiac calcific deposits and was in the range of 0 (no calcium visible) to 8 (extensive cardiac and aortic root calcific deposits).

**Table 1:** **Grading system of cardiac and aortic root calcium on echocardiographic examination**

Grade	Papillary muscle calcium	Mitral annular calcium	Aortic valve sclerosis	Aorta root calcium
**0**	Absent	Absent	Absent	Absent
**1**	Present	Mild **<**5 mm	Mild	Present
**2**		Moderate 5–10 mm	Moderate	
**3**		Severe **>**10 mm	Severe	

Aortic valve sclerosis graded as follows: Absent = Normal cusp thickness (<2 mm), and normal reflectivity; Mild = Cusp thickness >2 mm and/or increased reflectivity; Moderate = Thickness >4 mm and/or diffuse or focal cusp hyperreflectivity; Severe = Thickness >6 mm and/or marked echoreflectivity. Final score was graded from 0 to 8.

### Statistical analysis

Continuous variables are presented as mean ± standard deviation, if normally distributed, or median and interquartile range (IQR) if non-normally distributed; number and percentages are presented for categorical variables. CACS, nCACS score and eCS were not normally distributed, therefore we conducted our analyses using non-parametric tests. Furthermore, due to the non-linear relationship between the variables analyzed, we used Spearman's coefficient (rho) to test the correlation between 2 variables among eCS, CACS, nCACS scores and number of diseased vessels measured by CCTA. Receiver-operator characteristic (ROC) curves were used to evaluate the ability of the eCS and to identify the best cut-off to predict the presence of severe CACS (≥400) and obstructive CAD at CCTA in at least 1 vessel. In addition, ROC curve analysis was performed to evaluate the ability of nCACS and CACS to predict the presence of obstructive CAD. A two-tailed p value <0.05 was considered statistically significant. Statistical analyses were performed using Statsdirect ltd statistical software, Cheshire, UK.

## Results

### Clinical characteristics, eCS and CCTA data in the entire study population

Out of the 288 CCTA scans performed in the study period with an available evaluation of coronary artery tree, 147 patients were excluded because of either known CAD (n = 32), previous cardiac events (n = 45), evaluation of cardiac suspect neoplastic mass (n = 21), pacemaker leads creating artifacts (n = 3) or unavailability in our database of an echocardiogram performed within 3 months (n = 46).

A final study group of 141 subjects was produced. Median age was 67 y/o (IQR 58–75) with male gender representing 57% of the population; 66% were hypertensive, 55% hypercholesterolemic, 30% were active smokers and 14% diabetics.

Most subjects were intermediate-risk patients undergoing CCTA for evaluation of coronary artery tree; indications to CCTA were: suspected CAD in 118 patients (84%), planning of atrial fibrillation ablation procedure in 15 (11%) and other indications (risk stratification before aortic artery surgery, or dilated cardiomyopathy of unknown etiology) in 8 (5%). Using a >50% diameter stenosis to define obstructive CAD at CCTA, patients with no CAD, single-vessel, 2-vessel or 3-vessel disease were 86 (61%), 32 (23%),13 (9%) and 11 (7%), respectively; accordingly, 61% had no obstructive CAD, while 39% had at least one diseased (>50% stenosis) coronary artery.

**Figure 1:**
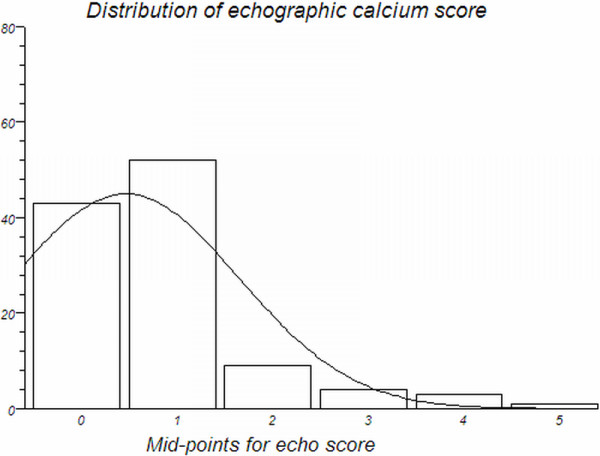
**Frequency distribution of echographic calcium score (eCS) in the study population, showing the skewed curve towards lowest scores.** Only one patient had a score = 6 (corresponding bar not visualized in the graph due to scale), while no patient had the most severe eCS of 7 or 8 points.

Among demographics and risk factors, only age and prevalence of hypertension differed significantly between the CAD and no CAD subgroups; while eCS, nCACS and CACS all differed significantly between the CAD and no CAD subgroups groups (Table 2). Figure 1 graphically depicts the frequencies distribution of eCS values in the entire study population.

**Table 2:** **Baseline demographics and clinical variables, echocardiography and computed tomography scores both in the entire population and in the subgroups with or without obstructive coronary artery disease**

	Entire population (n = 141)	CAD > 50% (n = 55)	No CAD > 50% (n = 86)	p
Age, median (IQR)	67 (58–75)	71 (61–80)	65 (55–73)	<0.01
Male gender (%)	80 (57%)	31 (56%)	49 (57%)	ns
Hypertension (%)	93 (66%)	46 (84%)	47 (55%)	<0.01
Family history of CAD (%)	47 (33%)	22	25	ns
Smoking (%)	43 (30%)	17	26	ns
Hypercolesterolemia (%)	77 (55%)	32	45	ns
Diabetes (%)	20 (14%)	11	9	ns
Obesity (%)	12 (8%)	4	8	ns
n. of vessels CAD > 50% (0-1-2-3)	86-32-13-11	-	-	-
eCS > 1	35 (25%)	22 (40%)	13 (15%)	<0.01
eCS, median, IQR, (range)	1, 0–1, (0–6)	1, 1–2, (0–6)	1, 0–1, (0–5)	0.015
CACS, median, IQR, (range)	29, 0–294, (0–3316)	294, 47–618, (0–3316)	3, 0–46, (0–1109)	< 0.001
nCACS, median, IQR, (range)	0, 0–97, (0–4377)	54, 0–178, (0–4377)	0, 0–65, (0–2936)	< 0.01

CAD = coronary artery disease, eCS = echographic calcium score, nCACS = multidetector computed tomography non-coronary artery calcium score, CACS = coronary artery calcium score, IQR = interquartile range.

### Reproducibility of eCS

A total of 40 echocardiographic exams were randomly selected and analyzed again 1 month later by a second observer to assess interobserver agreement for the eCS. According to weighted K test, interobserver agreement was good (weighted K = 0.78).

### Correlation data

Table 3 shows correlation data for all possible couples of variables among eCS, nCACS, CACS and the number of diseased vessels at CCTA.

**Table 3:** **Correlation data**

Correlation, rho, p value	nCACS	CACS	Number of vessels > 50%	Number of vessels > 70%
eCS	0.64, p < 0.0001	0.43, p < 0.0001	ns	0.28, p < 0.05
nCACS	-	-	ns	ns
CACS	0.51, p < 0.0001	-	0.44, p < 0.001	0.29, p < 0.05

eCS = echographic calcium score, nCACS = multidetector computed tomography non-coronary artery calcium score, CACS = coronary artery calcium score.

### Correlation between eCS and nCACS

The comparison between total eCS and the corresponding CCTA-derived cardiac score (nCACS), which was the primary aim of the study, yielded a positive and significant correlation (Spearman rho = 0.64, p < 0.0001), representing a strength of correlation usually defined as moderate or strong. Single components of the eCS or their partial combination correlated worse than the full eCS (AVC + MVC rho = 0.50, AVC rho = 0.49, aortic root calcium rho = 0.42, MVC rho = 0.32, p < 0.001 for all), or did not correlate as it was the case for papillary muscle calcification (p = 0.13).

### Correlation between eCS and CACS

Comparing eCS with the CACS also showed a significant positive correlation, but the strength of such correlation was inferior in magnitude compared to eCS and nCACS with rho = 0.46 (p = 0.0026), which is usually defined as moderate to low correlation.

### Correlation between either eCS, nCACS or CACS with CAD

When eCS was correlated with the number of diseased vessels at CCTA, there was a weak to low correlation (rho 0.28, p < 0.05) only when the most severe definition of CAD (diameter stenosis > 70%) was applied, while nCACS did not correlate with the number of diseased vessels, whatever the definition of obstructive CAD utilized. CACS correlated with the number of diseased vessels also (and more robustly than >70%) when the definition of milder disease, i.e. stenosis > 50%, was used. Only 12 pts had at least one >70% stenosis and consequently this definition of CAD was not further analyzed, due to lack of statistical power.

### Prediction of severe coronary calcification (CACS ≥ 400) and obstructive CAD

**Figure 2:**
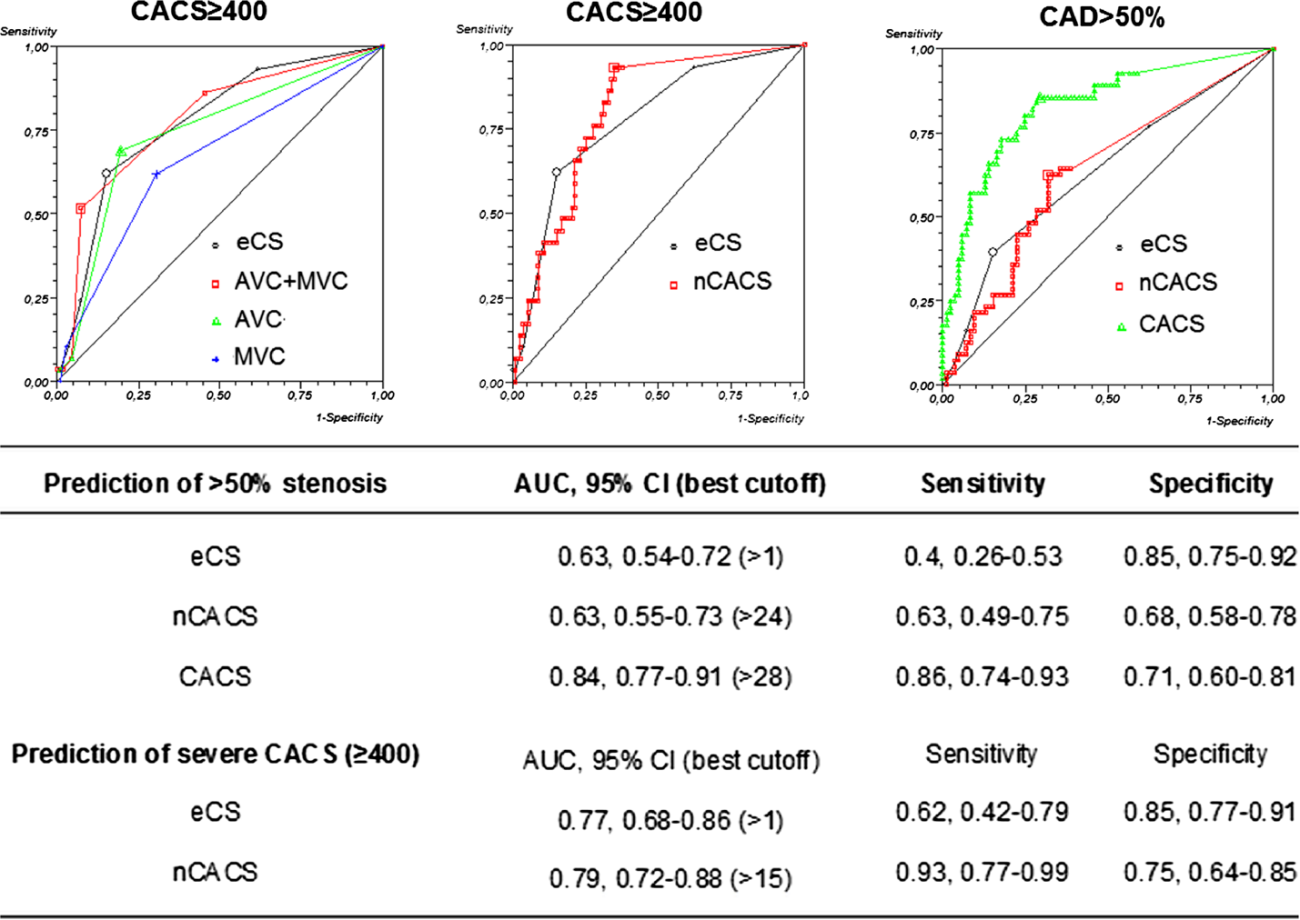
**Receiver operatore curve (ROC) plots for (left) the prediction of severe coronary calcification(CACS ≥ 400) by total echographic score (eCS), selected components (AVC or MVC) and their combination (MVC + AVC); best cutoff was >1 point both for eCS and combined AVC + MVC, while >0 for the separate components (either AVC or MVC).** Assessment of eCS and nCACS for severe coronary calcifications (CACS ≥ 400) (mid) or eCS, nCACSA and CACS for presence of obstructive CAD (right). AUC = Area under curve, AVC: aortic valve calcification, CACS = coronary artery calcium score calcifications, CAD = coronary artery disease, CI = confidence interval, eCS = echographic calcium score, nCACS = non-coronary artery calcium score.

ROC curves (Figure 2, upper part) demonstrate that eCS or AVC + MVC had similar capability (AUC 0.77 and AUC = 0.78, respectively) to predict a high CACS score, while AVC (AUC 0.74) and MVC (AUC 0.67) alone had slightly lower AUC values. Figure 2 (lower table) shows all AUC data and related best cutoffs for the prediction of either CACS ≥400 or obstructive CAD.

eCS and nCACS had similar accuracy for the prediction of both a high CACS ≥400 or obstructive CAD (at least one >50% stenosis) (Figure 2, lower part). Regarding the prediction of obstructive CAD, CACS had the highest predictive power when compared with both methods of cardiac *non-coronary* calcium assessment (nCACS and eCS).

**Figure 3:**
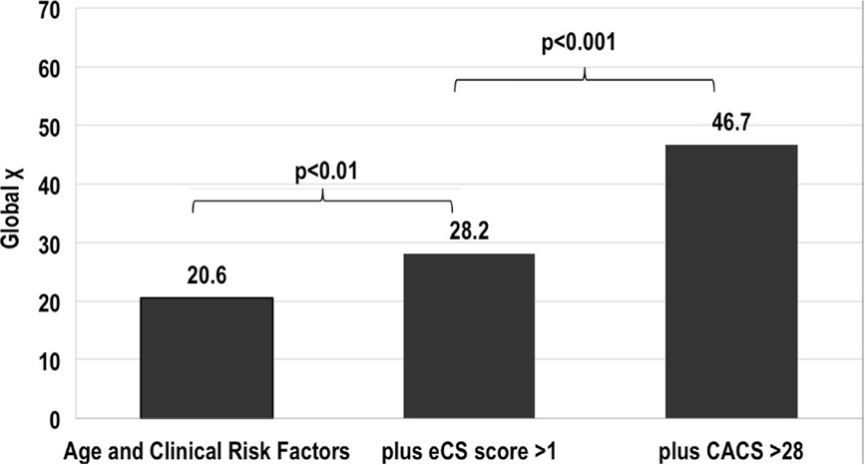
**Incremental value for the prediction of CAD > 50%.** Prediction of at least one coronary stenosis >50% at CCTA. Although the CACS score was the best addition on top of age, clinical risk factors and eCS, the addition of eCS was also a significant increasing step towards better prediction of obstructive CAD compared with starting clinical model. CACS = coronary artery calcium score calcifications, CAD = coronary artery disease, CCTA = coronary computed tomography angiography, eCS = echographic calcium score.

Figure 3 demonstrates that global chi square of logistic regression models significantly increased when adding eCS to age and clinical variables (global chi square 28.2 vs 20.6, p < 0.01) and further when finally adding CACS score (global chi square 46.7 vs 28.2, p < 0.001).

## Discussion

This study is the first to report data on eCS, nCACS, CACS and obstructive CAD in the same group of patients, whereas parts of these data were separately addressed in few previous studies [9, 10, 21].

The main finding in our study is that a calcium score (eCS), easily obtained from a standard echocardiographic examination is moderately to strongly correlated with cardiac *non-coronary* calcium score (nCACS). Furthermore, the full eCS score correlated better with nCACS than single components of the score itself (AVC, aortic root calcium, AVC + MVC and MVC). Finally, eCS also correlates with CACS and, in particular, it nicely predicts the presence of severe coronary calcification (CACS ≥ 400), a well-known predictor of future cardiovascular morbility and mortality, with a similar area under the curve compared with the corresponding non-coronary (nCACS) score by CCTA imaging. The eCS also significantly predicts the presence of obstructive CAD, and does so incrementally to age and clinical variables, but for this specific purpose, as expected, CACS remains more accurate.

### Breaking the calcium score into pieces

Although AVC, aortic root calcification or the combination of AVC + MVC correlated with total nCACS almost as well as full eCS, MVC was inferior for this purpose and papillary muscle calcification was instead not correlated at all. When analyzing the correlation of each single component of the eCS with the corresponding component of the nCACS (data not shown), the only component which did not significantly correlate between the 2 imaging methods was the papillary muscles calcium, which was scored as present in 20 patients by echocardiography and in no patient by CCTA. After reviewing those 20 cases, and confirming there were no significant errors on echocardiography or CTCA reading, the mismatch may be explained by the limitation of ultrasound in detecting the actual presence of calcium, relying mostly on thickening and hyperechogenicity, differently from CCTA specificity, thanks to the use of X-rays. Fibrosis of cardiac structures cannot be easily differentiated from calcification using conventional ultrasound imaging and this may possibly account for differences between echocardiography and CCTA in papillary muscles calcification data, which we hypothesize in fact do represent fibrosis and not calcification.

Papillary muscles could probably be eliminated from the eCS, without decreasing the predictive accuracy of the score itself, although the number of patients in our study who demonstrated papillary muscles calcification using ultrasound was too low to draw conclusions.

### Cardiac calcifications for either diagnosis of CAD or prognosis

From a clinical standpoint, a wealth of previous literature regarding eCS or MVC or AVC assessment convincingly demonstrates the *prognostic value* of ultrasound calcium assessment [11–19], compared with the more elusive diagnostic prediction of obstructive CAD [8–10, 24, 25], for which clinical purpose other tools, such as CCTA or provocative testing, may be more useful. One previous study [9], conducted on the same number and type of patients, but lacking nCACS data, confirmed the relation between eCS, CAC and CAD, but found that the best predictive cutoff was eCS > 2, while in our study the best cutoff was one point lower (eCS > 1). This confirms the presence of a grey zone, as in most scores in medicine, between eCS 1 and 2, in which results should be interpreted with clinical caution. Our study confirms that ultrasound-detected cardiac calcium, assessed with a simple but comprehensive score, encompassing left heart valves, aortic root and papillary muscles, does predict obstructive CAD similarly to nCACS, though the clinical usefulness for CAD diagnostic purpose is limited by its moderate accuracy, compared with CACS. On the other hand, our study confirms that echocardiographic cardiac calcium moderately correlates with coronary calcium and its prediction of cardiac events, at least in part, works through prediction of coronary artery stenosis. Other mechanisms can be hypothesized: in fact, published data from CCTA studies showed that the presence of cardiac calcium *outside* the coronary tree may not only reflect coronary calcium burden [5, 6] but also the vulnerability of atherosclerotic plaques, acting like a feature of additional risk for cardiac events, independently from the presence or grade of a coronary stenosis [7]. This would explain the robust link between cardiac calcium and prognosis, in spite of a weaker correlation with coronary artery stenosis grade. A recent study linked coronary plaque vulnerability to calcium density in the plaque, by so doing demonstrating that the total burden of coronary calcium is only part of the cardiac risk picture [26].

### What clinical use for eCS?

A hypothetical practical clinical use for the eCS, by virtue of its higher specificity compared with (lower) sensitivity value for severely calcified coronaries, which in turn predict both obstructive CAD and coronary events, would be to reclassify patients at estimated low or intermediate clinical cardiac risk, to a higher risk class in those cases in which eCS is >1 or, even better (to incorporate the best cutoff of a previous similar study), eCS > 2. In fact, when raising the cutoff from >1 to >2 points in our study the specificity of eCS to predict CAD went from 85% up to 93%, but at the obvious expense of sensitivity becoming trivial (16%). This hypothesis clearly needs prospective validation, which we are currently implementing in a wide prospective outcome study.

### Strengths and limitations

**Figure 4:**
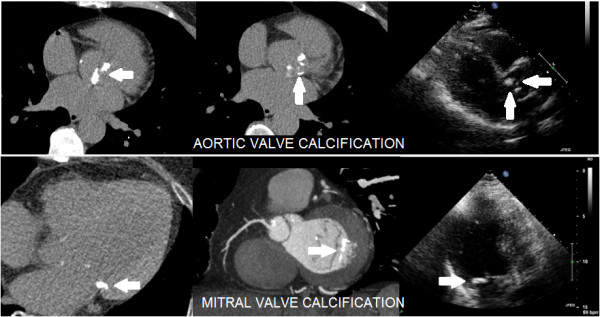
**Computed tomography (left and mid) and echocardiography (right) parallel imaging of calcifications.** Upper panel shows aortic valve calcification and lower panel shows mitral valve calcification.

This study is a single-center and retrospective one, potentially subjected to patient selection bias. The retrospective identification of patients having both an echocardiogram and CCTA available forced us to accept the quality of available echocardiograms, sometimes incomplete for an ideal calcium assessment; this may be regarded as a drawback or as a strength of our study, using “real-life” standard echocardiograms. Comparison between a semi-quantitative and a fully quantitative approach is difficult, and the eCS requires some operator experience to control gain settings in order to obtain reproducible results; thus, CCTA remains a more objective approach in this regard (Figure 4 shows examples of cardiac calcifications imaged both at CCTA and echocardiography). It is not always possible to distinguish sclerosis from calcification, which probably represent two phases of the same pathophysiologic process; accordingly, in our study sclerotic lesions were included along with calcified lesions in the eCS. The non-simultaneity of ultrasound and CCTA examinations is probably a minor drawback, since the 2 tests were per protocol separated by less than 3 months, a trivial interval for the chronic atherosclerotic process.

In 2011 Pressmann et al. [21] reported comparable strength of correlation, in a smaller group of similar patients with clinical indication to cardiac computer tomography, between their ultrasound score and either nCACS (rho 0.56 vs 0.64 in our study) or CACS (rho 0.46 vs 0.43 in our study), confirming the trend towards better correlation with nCACS rather than CACS, which cannot be directly visualized by ultrasound. Our study is the first using the latest technology, 128-slice dual-source scanner for CCTA, which is the most accurate non-invasive technology to assess obstructive CAD and minimizes radiation exposure.

## Conclusions

This study demonstrates that cardiac non-coronary calcium by echocardiography correlates well with the same measure by computed tomography, with severe coronary artery calcification and, although less strongly, with obstructive coronary artery disease.

Echocardiographic calcium score, which is now robustly validated against non-coronary calcium by computed tomography, might be used as a calcium score to be widely utilized at low cost and with no irradiation safety issues, on top of clinical risk score, for individualized refinement and potential reclassification of cardiac risk. This use needs to be prospectively tested in multicenter outcome studies, investigating the risk reclassification potential of eCS, which could possibly optimize the use of known disease-modifying therapies, such as statins.
